# The Functionality of Inulin as a Sugar Replacer in Cakes and Biscuits; Highlighting the Influence of Differences in Degree of Polymerisation on the Properties of Cake Batter and Product

**DOI:** 10.3390/foods10050951

**Published:** 2021-04-27

**Authors:** Kleopatra Tsatsaragkou, Lisa Methven, Afroditi Chatzifragkou, Julia Rodriguez-Garcia

**Affiliations:** Department of Food and Nutritional Sciences, University of Reading, Whiteknights, Reading, Berkshire RG6 6AD, UK; kleotsat@gmail.com (K.T.); l.methven@reading.ac.uk (L.M.); a.chatzifragkou@reading.ac.uk (A.C.)

**Keywords:** cake, biscuit, sugar, inulin, degree of polymerisation, rheology, texture, sensory perception

## Abstract

Sugar has multiple roles in baked products; competing for water and as such reducing starch gelatinisation and gluten development, behaving as a fluid during cooking, recrystallising on cooling; roles which influence properties such as aeration, texture and mouthfeel. Partial replacement with inulin, can provide beneficial nutritional and functional properties. This paper investigated the degree of polymerisation (DP) of two commercial inulins and their influence on baked product properties as a 30% sugar replacer. The two inulins varied substantially in their proportion of longer fructans (32.7% compared to 17.5% of DP > 11). The lower DP inulin led to a cake batter with very similar viscoelastic properties to the standard sugar batter, and subsequently to a very similar baked cake crumb structure, cake texture and mouthfeel. The higher DP inulin led to a more viscous batter, and cake with a less homogenous crumb structure that was perceived to be dryer and more mouthcoating. The subsequent use of the lower DP inulin in a biscuit formulation resulted in a slightly less elastic dough and consequently a softer and less crunchy biscuit. In summary, the success of inulin in providing functional properties that can enable sugar reduction in baked products is dependent on the degree of polymerisation of the inulin and rheological parameters needed in the specific bakery matrix.

## 1. Introduction

Increasing public awareness for a healthier diet, alongside World Health Organization reports stating that one in three adults worldwide was overweight in 2008 [1], has led the food industry to focus on the production of reduced sugar foods with physical and sensory properties comparable to those of conventional products. Sweet bakery products such as cakes and biscuits are a regular part of today’s diet. Biscuits are very popular bakery products, because of their availability in different varieties and at an affordable cost [2]. Cakes too have high consumer acceptance providing a generally softer texture than biscuits. Their common feature is the use of sucrose as one of the main ingredients. In biscuits, sucrose affects flavour, dimension, colour and texture [3]. Similarly, in cakes, the role of sucrose extends beyond providing sweetness; it affects the physical structure of cakes by regulating the gelatinisation of starch. The delay in starch gelatinisation during baking allows air bubbles to expand as the vapour pressure increases from carbon dioxide and water vapour, allowing the formation of a voluminous cake structure [4].

The effect of different sugar substitutes such as natural and artificial high-intensity sweeteners, fructans and bulking agents such as polyols on sweet-baked product characteristics have been assessed by various researchers. Inulin is a soluble fibre that can be added into sweet bakery product formulations to achieve healthier nutritional profiles, such as sugar reduced, fat reduced and calorie reduced products. There are a number of studies in the literature concerning the use of inulin for fat replacement to enhance the dietary value of cakes and biscuits. However, the use of inulin as a sugar replacer in cakes and semi-sweet hard biscuits, as investigated in this study, is not widely reported [5]. The most recent publications concerning the use of inulin for sugar reduction in cakes focus on the effect of inulin on the glycaemic index (GI) of cakes or on their microstructure [6,7]. Struck et al. [8] reported the use of different fibres combined with rebaudioside A for the production of sugar-reduced muffins. The authors concluded that the crumb firmness and the crust colour lightness increased at the lower sucrose levels. However, the application of wheat fibre at 30% sugar reduction level gave a sugar reduced muffin with density and volume closest to the reference. According to Psimouli and Oreopoulou [9], sugar-reduced cakes prepared with oligofructose, lactitol and maltitol presented no significant differences from the control cake in terms of rheological behaviour, specific volume of the end-product, textural and most sensory properties. Concerning biscuits, Canalis et al. [10] and Mancebo et al. [11] recently studied the effect of adding inulin on short dough biscuits for fibre supplementation. Giarnetti et al. [12] and Krystyjan et al. [13] studied the use of inulin as a fat replacer in short-dough biscuits. Mieszkowska and Marzec [14] reported the effect of inulin as a sugar replacer on the texture and sensory properties of short-dough biscuits and Laguna et al. [15] proposed that inulin could be used to replace up to 25% of sucrose in short-dough cookies without having a detrimental effect on consumer perceptions of the product. The overall conclusion from these studies is that inulin can be used successfully to replace fat or sugar in biscuits and cakes if applied at the appropriate level as the reformulated products exhibited similar textural and sensorial characteristics to control samples. However, it is very important to understand the effect degree of polymerisation (DP) of inulin on product physicochemical and sensory properties, in order to select the most appropriate ingredient for sugar replacement in each formulation, to match the full sugar products. To the best of our knowledge, such information is limited in the literature.

The present work focused on determining the effect of two commercially available inulin ingredients with different degrees of polymerisation on the rheology, physical and sensorial properties of cakes where sugar was reduced by 30%. The hypothesis of this study was that the DP of commercial inulin ingredients would vary and that this would have a significant and substantial effect on cake properties. The inulin product resulting in a final cake product most closely matching the full sugar control was then further tested in a semi-sweet biscuit product. This study aims to increase understanding of the functionality of inulin as a sugar replacer within the different processing steps of cake and biscuit manufacturing. This knowledge is essential in order to improve the quality of sugar reduced biscuits and cakes and develop products equivalent to the traditional full sugar products on the market.

## 2. Materials and Methods

### 2.1. Materials

The ingredients used in the preparation of cakes were a cream cake premix without sugar (protein 9.7%, starch 66% on dry basis; Puratos, Dilbeek, Belgium), whole liquid pasteurized eggs (Brakes, Kent, UK), granulated sugar (Tate & Lyle, London, UK) and rapeseed oil (purchased from a local UK supermarket).

The ingredients used in the preparation of biscuits were soft wheat flour (moisture content 11.9%, protein 9.3%, dietary fibre 2.7%, lipid 0.98%), fat (palm oil/canola oil mixture), inverted sugar, rapeseed lecithin, white granulated sugar, salt, sodium bicarbonate, ammonium bicarbonate, sodium metabisulfite and sodium acid pyrophosphate (SAPP 28).

The sugar replacers used in batters and cakes were: highly soluble inulin powder Orafti^®^ HSI (Beneo GmbH, Mannheim, Germany) with a degree of polymerization (DP) between 2 and 60, inulin content minimum 88% and glucose/fructose/sucrose content 12% (kindly donated from Kreglinger Ltd., Chesham, UK) and native inulin Fibruline^®^ Instant (Cosucra, Pecq, Belgium) with a DP of approximately 10, a minimum inulin content of 90% and glucose/fructose/sucrose content 10%, kindly donated by Caldic Ltd. (Chesterfield, UK). Just one sugar replacer was used in doughs and biscuits, that of highly soluble inulin powder Orafti^®^ HSI (Beneo GmbH).

The 0.1 M sodium hydroxide and 1.0 M sodium acetate solutions used in the ion chromatography were made of electrochemical grade sodium hydroxide (50%, w/w, ThermoFisher, Loughborough, UK) and sodium acetate (ThermoFisher), prepared with high quality deionized water of low resistivity (18 MΩ) (Milli-Q direct, Merck, Gillingham, UK).

### 2.2. Analysis of Polymerisation of Inulin Fibres by Ion Chromatography

Orafti^®^ HSI and Fibruline^®^ Instant samples were dissolved in purified water and were subjected to high-performance anion-exchange chromatography with pulsed amperometric detection (HPAEC-PAD), using ICS-MS 6000 (ThermoFisher). Separation was carried out on a Dionex Carbopac PA1 column (10 μm, 4.0 × 250 mm) (Thermo Scientific, Loughborough, UK), at 30 °C. A gradient analysis was performed using (A) 0.1 M sodium hydroxide and (B) 1.0 M sodium acetate/0.1 M sodium hydroxide eluents. Running conditions were 0–15 min: 100 mM NaOH/20 mM NaOAc, 15–70 min 100 mM NaOH/20–450 mM NaOAc, 70–70.1 min: 100 mM NaOH/20 mM NaOAc, 70.1–75 min: 100 mM NaOH/20 mM NaOAc), at a flow rate of 1 mL/min and an injection volume of 10 μL. Chromeleon 7.2 software (Thermo Scientific) was used to interpret the chromatograms. The relative percentage of DP was calculated based on the ratio of each peak area to the total sum of integrated peak areas for each sample. The DP of samples was identified based on in-house inulin hydrolysates originating from Jerusalem artichoke inulin samples that were used as reference standards.

### 2.3. Cake Preparation

The basic recipe for the cake batter was: 540 g cake premix, 460 g sugar, 350 g pasteurised whole liquid egg, 225 g water and 300 g rapeseed oil. In sugar reduced formulations, inulin replaced sugar at 30% w/w. The control cake contained 27% sugar, and the two sugar reduced products contained 19% sugar. Cake batter was prepared in a Kenwood Major mixer (Kenwood Ltd., Havant, UK). All ingredients were added together and mixed using a flat beater for 2 min at speed 2 (low speed) and 2 min at speed 4 (high speed). Required quantities of batter (450 g) were placed into aluminium baking trays and baked at 180 °C in a rotary oven for 40 min. After baking, cakes were removed from the trays, cooled (1 h, 20 °C) and subjected to physical tests the same day of production. Cake samples for sensory evaluation, were sealed in polyethylene bags, and frozen at −18 °C. They were defrosted at room temperature the day before each sensory session. Resulting products were named as follows: control (full sugar cake, no inulin), RS Orafti (30% sugar reduced cake with Orafti^®^ HSI), RS Fibruline (30% sugar reduced cake with Fibruline^®^ Instant). All formulations were prepared in triplicate.

### 2.4. Biscuit Preparation

The biscuits used in this study were a hard dough type where the gluten network is developed further than in short-dough type of biscuits. The basic recipe for the biscuit dough was 100 g flour, 17 g fat, 25 g sugar, 25 g water, 4.5 g inverted sugar, 0.25 g rapeseed lecithin, 0.4 g salt and 1.1 g leavening agent. The baked full sugar control biscuit contained 22% sugar, whereas the sugar reduced product contained 15%. The main dough ingredients mixed in a z-blade mixer (Morton Mixers, Bellshill, UK) for 1 min at low speed (48 rpm), with water added last. The bowl was scraped down, sodium metabisulfite and SAPP 28 were added, and the mixture was mixed again for 1 min at low speed (20% input AVI). Mixing was carried out at a higher speed (84 rpm) for 12 min until the dough temperature reached 40 °C The dough rested for 10 min (38 °C) before sheeting (Rondo, Chessington, UK) to 20 mm thickness. Biscuits were cut (70 × 55 mm rectangle), placed on a perforated tray and baked in an electric fan oven (Kwik-co, Salva, Spain) for 8 min at 180 °C. After cooling (30 min, 20 °C) biscuits were stored in sealed polyethylene bags at room temperature. Resulting products were named as follows: control (full sugar biscuit, no inulin), RS Orafti (30% sugar reduced biscuit with Orafti^®^ HSI inulin). All formulations were prepared in triplicate.

### 2.5. Rheological Methods

Rheological properties of the cake batters and biscuit doughs were studied using an oscillatory rheometer (MCR 302, Anton Paar Ltd., St Albans, UK) using parallel serrated plate geometry (50 mm diameter for viscosity and frequency sweep tests, 25 mm diameter for the temperature sweep, profile 1 mm x 0.5 mm) in order to prevent sample slippage. The gap size was 1 mm for cake batter and 1.8 mm for biscuit dough. For biscuit dough cylindrical samples were taken from the sheeted dough (20 mm height) using a cylindrical cutter of 6 cm diameter. Experiments were performed in duplicate for each of the three process batches, resulting in a total of 6 replicate measurements per formulation.

For cake batters apparent viscosity was measured as a function of shear rate over the 0.1 to 100 s^−1^ range, at 25 °C. Data were fitted to the following power law (Ostwald model):

η = K·γ^(n−1)^ (1)

where η is the viscosity (Pa·s), γ is the shear rate (s^−1^), K is the consistency index (Pa·sn), and n is the flow behaviour index. For a Newtonian fluid the flow behaviour index = 1; for a shear-thinning fluid it is between 0 and 1 and for a shear thickening fluid it is greater than 1.

In both cake batter and biscuit dough, a frequency sweep test was performed with frequency varying from 0.1 to 100 rad/s at 0.05% strain (in the viscoelastic linear regime) and at 25 °C for cake batters and 35 °C for biscuit dough (dough temperature when sheeted at industrial scale). Dynamic rheological properties (mechanical spectra) of samples were recorded by monitoring the shear storage modulus G′, characterizing the elastic behaviour of the sample, and the shear loss modulus G″, describing the viscous behaviour of the sample. Loss factor, tan δ, was calculated according to the following equation:

tan δ = G″/G′ (2)

Temperature sweep tests were performed from 25 °C to 160 °C for cake batters (35 °C to 160 °C for biscuit dough) at a heating rate of 2.5 °C/min for batters (5 °C/min for biscuit dough) and a strain amplitude of 0.05% in order to simulate the effect of baking. The storage modulus (G′), the loss modulus (G″) and the complex shear modulus (G*) were recorded. The complex shear modulus was calculated as G* = G′ + i G″. In this study the thermal setting temperature (TST) was calculated according to the method proposed by Sikora et al. [16]. The first derivative of the curve (dtanδ/dt) was calculated, and the inflection points were identified, which precisely corresponded to the TST of the systems.

### 2.6. Physical Property Measurement of Products

#### 2.6.1. Weight Loss during Cake Baking

Percent weight loss (WL (%)) of cakes during baking was calculated by using the mass of cake batter (W0) and mass of cake sample just after baking (W1):

WL(%) = [(W0 − W1)/W0] × 100(3)

For each batch to batter the correspondent cakes were weighted; a total of 12 replicate measurements per formulation.

#### 2.6.2. Water Activity and Moisture Content

Moisture content of cake and biscuit crumb was measured using Sartorius M-Pact Series (Sartorius Lab Instruments, Goettingen, Germany). From each cake, two slices were crumbed and 6 g of crumb mix were placed on an aluminium tray and heated at 150 °C until constant weight. Biscuits were crumbled and 3 g of crumb were placed on an aluminium tray and heated at 129 °C until constant weight. Water activity was measured using a HygroLab (Rotronic Instruments, Crawley, UK); 2 g of crumbed sample was used. For cakes two loaves were tested from each of three process batches, and two slices per loaf (resulting in 12 replicate measurements per formulation). For biscuits, an average was taken from 10 biscuits per formulation.

#### 2.6.3. Colour Measurement

Colour measurements were carried out using Chroma Meter CR-400 colorimeter (Konica Minolta, Warrington, UK). For cakes, the colour of the crumb was measured in 3 different regions of a cake slice, from one slice per cake, and two cakes of 3 batches (12 replicate measurements per formulation). For biscuits, measurements were taken at the middle of the top surface; 3 biscuits per batch were measured. Results were expressed in relation to the L*, a* and b* parameters of the CIELAB system (illuminant C and 10° viewing angle). The L* value relates to the lightness and it ranges between 0 (black) and 100 (white), a* varies between −a* (greenness) and +a* (redness) and b* ranges between −b* (blueness) and +b* (yellowness).

#### 2.6.4. Cake Height and Cellular Structure of the Crumb

Cakes were cut in slices of 1.5 cm thickness, vertically with a slicing machine (Crypto Peerless S300, Halifax, UK). The cut side of each slice was scanned (HP Scanjet G2710) with a resolution of 300 dpi. The cake height was measured at the centre point from the cross section of the product using Image J (National Institutes of Health, Palo Alto, CA, USA). The crumb cell structure of the scanned images was analysed using the software ImageJ: a 3.5 × 3.5 cm section was cropped from the central part of each slice, on which the analysis was performed. First, the image was split into colour channels, then the contrast was enhanced and, finally, the image was binarised after grayscale threshold. Mean cell area (mm^2^), cell circularity and cell density (cells/cm^2^) were calculated. The circularity was calculated using the formula: circularity = 4π (area/perimeter^2^) and the values of circularity range between 0.0 to 1.0; a circularity value of 1.0 indicates a perfect circle, and as the value approaches 0.0, it indicates an increasingly elongated polygon. Measurements were conducted using 2 slices from the centre of each cake, 2 cakes for each of 3 batches (resulting in 12 replicate measurements per formulation).

#### 2.6.5. Biscuit Dimensions

Length, width and thickness (mm) of the biscuits were measured using a digital caliper, taking an average from 20 biscuits of each formulation.

#### 2.6.6. Texture Analysis

Texture analysis was performed using a TAXT-Plus Texture Analyzer (Stable Micro System, Godalming, UK). Cake slices (25 mm thickness each) were cut from the centre of the loaf and a uniaxial compression test with subsequent relaxation phase was applied in order to evaluate the firmness and springiness of the crumb. A 38 mm diameter cylindrical probe, a test speed of 1.0 mm/s and at 25% strain deformation were used. The sample was initially compressed at 25% of its height, then the probe holds at this distance for 60 s (relaxation phase) and then withdraws from the sample. Firmness was taken as the force value required to compress the sample by 25% of its height. Springiness of the crumb (the force with which the crumb resists the defined mechanical stress during compression) was derived from the recorded force–time diagram. The calculation was done according to the following equation:

Springiness % = (Fres/Fmax) × 100(4)

where Fmax is the maximum force at 25% compression of the crumb and Fres is the residual force after 60 s of relaxation phase. Measurements were performed in from 2 slices per cake, and two cakes of 3 batches (12 replicate measurements per formulation).

The hardness and stickiness of the biscuit dough were assessed using a sphere penetration test. A stainless-steel spherical probe (1.27 cm diameter) and a 5 kg load cell were used. 10 dough discs were taken from the sheeted dough (20 mm height) using a cylindrical cutter of 6 cm diameter from each batch. The penetration test was performed at a pre-test, test and post-test speed of 1.5, 1.0 and 10 mm/s, respectively and at a 60% strain. Penetration was performed on the centre of the sample and a trigger force of 5 g was used. Three separate batches of biscuit dough were prepared and in each case 10 dough disks were measured.

The fracture properties of biscuits were evaluated using a resistance to bend test; biscuit firmness was determined by the bend or ‘‘snap’’ test, using the 3-point bend rig (settings: pre-test speed, 2 mm/s; test speed, 1 mm/s; post-test speed, 10 mm/s). The biscuit was centered on two parallel adjustable supports of the rig base plate at a 5 cm distance apart. Each biscuit was compressed once, and peak force was recorded. 10 biscuits were tested from each formulation.

### 2.7. Sensory Evaluation: Quantitative Descriptive Sensory Analysis (QDA)

A trained sensory panel (n = 10–12), with a minimum of two years’ experience, developed consensus descriptive vocabularies of the sensory attributes (appearance, aroma, taste/flavour, mouthfeel and aftereffects) over three training sessions for cakes and three for biscuits, using reference standards to assist in defining attributes where required (Supplementary Table S1). For cake, approximately 25 g of each sample (30 × 30 × 60 mm), with crust removed, were presented to panellists at each tasting on a 3-digit coded ceramic grey plate. For biscuits, approximately 3.5 g of each sample (half of a biscuit, 50 × 35 mm), were presented at each tasting to panellists on a 3-digit coded ceramic grey plate. During duplicate quantification, samples were presented in a balanced monadic sequential order and sample attributes were scored by panellists individually on visual analogue scales (scaled 0–100) using Compusense cloud software (Compusense Inc., Guelph, ON, Canada). In order to minimize carryover effects, a 1 min interval was allowed between each sample. In addition, panellists were provided with filtered water and asked to cleanse their palate between tastings. Panellists received the agreed list of attributes, including definitions, to aid them during sample evaluation. Unstructured lines scales were used except for sweetness where a structured scale was used with 5 reference anchors. The reference anchors were 5 aqueous sucrose solutions of increasing sweetness (2, 3, 4, 5 and 6% w/v) for which the anchor positions had been agreed by the panel (anchor point 1 [low] = 0, 2 = 25, 3 = 50, 4 = 75 and 5 [strong] = 100)). These standards were given to the panellists at the beginning of the test for refamiliarisation when panellists were instructed to score the sweetness on the structured line scale. All assessments were carried out in isolated sensory booths under artificial daylight and with the room temperature controlled at 23 °C.

### 2.8. Statistical Analysis

Analysis of variance (ANOVA) on the data was performed using the XLSTAT software package (Addinsoft, Paris, France). Multiple pairwise comparisons using Tukey’s HSD test, were used to evaluate mean values’ differences (*p* < 0.05).

Sensory data analysis was performed using SENPAQ (version 5.01; QI Statistics, West Malling, UK) using two-way ANOVA, with sample fitted as a fixed effect, panellists as a random effect and effects tested against the sample by panellist interaction. Significant differences between samples were assessed by Fisher’s LSD post hoc tests. Principle component analysis (PCA) was based upon the covariance matrix.

## 3. Results and Discussion

### 3.1. The Distribution of Degree of Polymerisation (DP) of Inulin Samples

**Figure 1:**
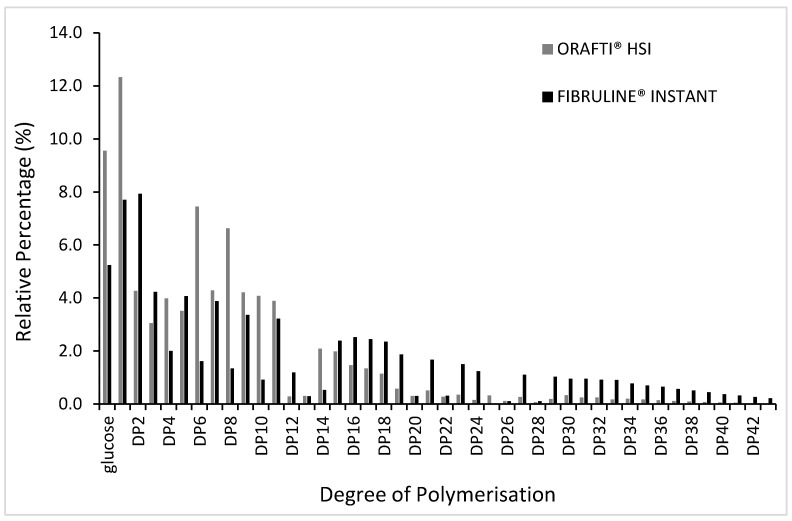
Degree of polymerisation (DP) distribution of Orafti^®^ HSI inulin (grey bars) and Fibruline^®^ Instant inulin (black bars), analysed by ion chromatography.

The two commercial inulin fibres differed in their DP (Figure 1). Both commercial fibres are reported to contain low amounts of mono and disaccharides (less than 12% glucose/fructose/sucrose content for Orafti^®^ HSI and less than 10% for Fibruline^®^ Instant); this was indeed found to be the case (Figure 1) with the relative percentage of glucose being just under 10% and 6%, respectively. Fibruline^®^ Instant as a native inulin had longer chain lengths and a higher DP. It is sold with a DP of approximately 10 and the results here concluded an estimated fructooligosaccharides (FOS) distribution (DP 3–10) accounting for 21.4% and fructans (DP > 11) accounting for 32.7%. In comparison, the highly soluble Orafti^®^ HSI contained a higher proportion of FOS (37.2%) and a much lower percentage of the longer fructans (17.5%).

### 3.2. The Influence of Inulin as a Sugar Replacer in Cakes

#### 3.2.1. Batter Viscosity

Batter viscosity is a crucial parameter for cake final quality characteristics and especially for volume. Batters with very low viscosities cannot hold air bubbles in their matrix and cakes collapse in the oven, whereas a highly viscous batter can restrict its expansion during baking [17,18].

**Figure 2:**
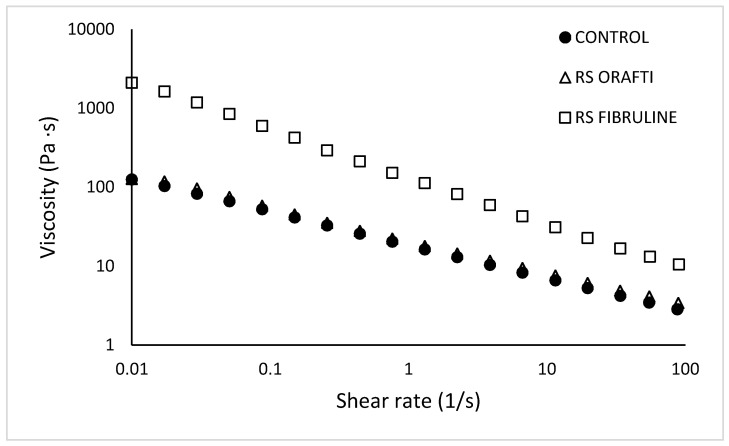
Effect of inulin type and sugar content on flow properties of cake batters at 25 °C. Control (full sugar cake batter), RS Orafti (30% sugar reduced cake batter with Orafti^®^ HSI inulin), RS Fibruline (30% sugar reduced cake batter with Fibruline^®^ Instant inulin). The data are mean values of replicates.

Apparent viscosity values versus shear rate are presented in Figure 2. A decrease in viscosity with increasing shear rate can be observed for all samples indicating a shear thinning behaviour. A power law equation was found to adequately describe the experimental data (R^2^ > 0.99); the consistency index (K) and flow behaviour index (n) for the different formulations are presented in Table 1. The type of inulin had a significant effect on the viscosity of the cake batters; Orafti^®^ HSI inulin resulted in batter viscosity values similar to the control (4.13 and 3.47 Pa·s at 54 s^−1^, respectively), whereas Fibruline^®^ Instant significantly increased viscosity (13.23 Pa·s at 54 s^−1^), showing a significantly higher (*p* < 0.05) K values than control and RS Orafti samples (130.09, 18.48 and 20.79 Pa·s^n^, respectively). These changes are likely to result from different degrees of polymerization, difference in molecular weight, and size distribution of the different fractions of both inulin types [19]. Indeed, Fibruline^®^ Instant did have longer chain lengths and, a higher DP (32.7% of fructans; Section 3.1) than the Orafti^®^ HSI (17.5% of fructans; Section 3.1) which does explain the higher batter viscosity. Moreover, RS Fibruline batter showed a lower (*p* < 0.05) flow behaviour index (n) than the other two samples (0.38 vs. 0.58), indicating that this batter had stronger pseudoplasticity. Fibruline^®^ Instant had higher DP, thus the flexibility of the molecules is lower for smaller molecules, such as Orafti^®^ HSI, showing a restricted motion at lower shear rates. The lower water solubility of Fibruline^®^ Instant in comparison to Orafti^®^ HSI, also explains its stronger pseudoplastic behaviour; Fibruline^®^ Instant was present mainly as dispersed molecules that were deformed and rotated, showing less resistance to flow as shear rates increased. In contrast, Orafti^®^ HSI molecules were highly tighten to water, and the smaller molecules were probably solubilized, so this batter showed less resistant to flow (lower K) and less shear rate dependent behaviour (higher n). Similar results were found by Kou et al. [20] when adding Fibruline^®^ Instant to a wheat starch dispersion at concentrations higher than 5%.

#### 3.2.2. Viscoelastic Properties of Cake Batters

**Figure 3:**
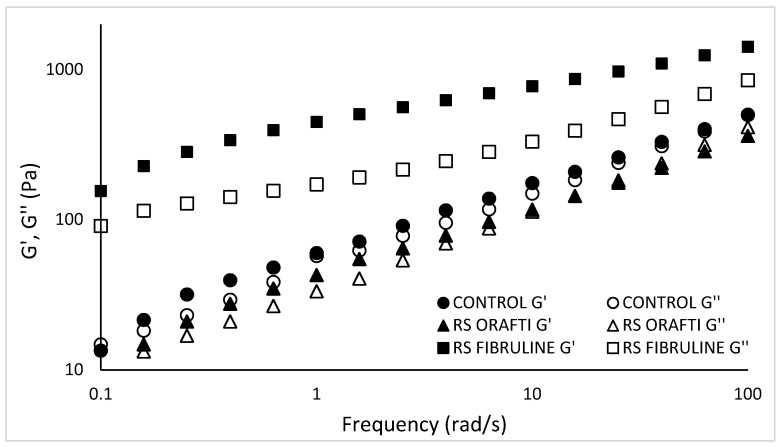
Storage modulus (G′) and loss modulus (G″) of cake batters. Control (full sugar cake batter), RS Orafti (30% sugar reduced cake batter with Orafti^®^ HSI inulin), RS Fibruline (30% sugar reduced cake batter with Fibruline^®^ Instant inulin). The data are mean values of replicates.

The frequency sweep describes the viscoelastic properties of the batter that result from the arrangement of its constituents and hence the microstructure of the batter. Figure 3 demonstrates the dynamic oscillation data curves G′ (storage modulus) and G″ (loss modulus). Both G′ and G″ showed a frequency dependence revealing a typical rheological behaviour of weak gels. This frequency dependence was more obvious for control and RS Orafti batters than for RS Fibruline batter. In general, RS Fibruline viscoelastic moduli were higher than RS Orafti and control batter (G′ at 10 rad/s: 775.34 vs. 117.16, 175.79 Pa, respectively and G″ at 10 rad/s: 0.43 vs. 0.98, 0.85 Pa, respectively). The viscoelastic properties of batters are strongly influenced by the molecular weight of the ingredients on them. The higher DP of Fibruline^®^ Instant inulin corresponded to batters with higher G′ and G″, since longer chains gave place to higher number of entanglements in the system. All samples had a higher G′ than G″ curve indicating a higher elastic character over viscous; however, this is more the case for the RS Fibruline batter than the RS Orafti and control batters, which showed a more fluid like character. The results also show (Figure 3) that control and RS Orafti batters behaved as relatively weak gels, as they showed a cross over point at the high frequency end of the spectrum; RS Orafti batter exhibited a G″ > G′ around 15 rad/s, while control exhibited a crossover point at higher frequencies, around 100 rad/s. 

The frequency crossover point tends to increase with decreasing the molecular weight, corresponding to shorter time necessary for the polymer chains to disentangle [21]; RS Orafti batter showed a lower frequency crossover point as the high soluble inulin had a higher DP than the sucrose present in the control batter. Similar results were observed by Bruno et al. [22], when comparing the viscoelastic properties of ethyl cellulose samples of different molecular weights prepared into gels.

#### 3.2.3. Rheological Properties of Batters during Heating

**Figure 4:**
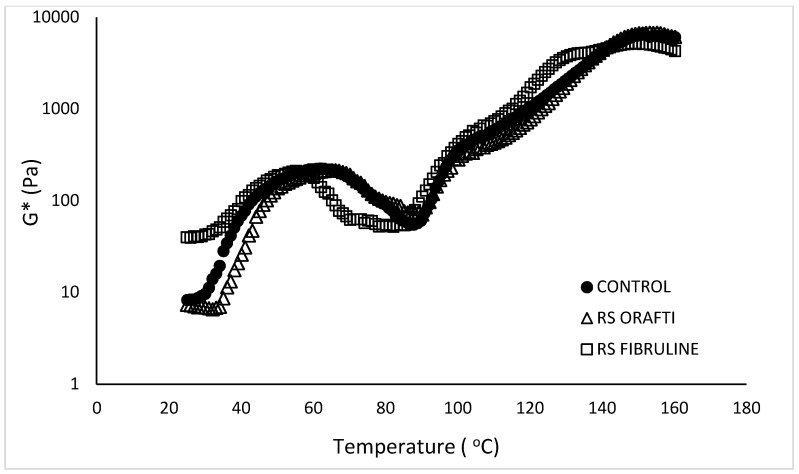
Complex modulus G* as a function of temperature of cake batters. Control (full sugar cake batter), RS Orafti (30% sugar reduced cake batter with Orafti^®^ HSI inulin), RS Fibruline (30% sugar reduced cake batter with Fibruline^®^ Instant inulin). The data are mean values of replicates.

To investigate the structural changes taking place in the different cake batters during heating, viscoelastic properties were studied from 25 °C to 160 °C, aiming to simulate the batter’s behaviour in the oven. The structural changes that occur in the batter during baking determine bubble formation, stability and the final baked product’s structure and texture [23]. In particular, the role of sucrose is crucial during heating as it increases the starch gelatinisation and protein denaturation temperatures. With an increase in starch gelatinisation temperature, the change of batter from a fluid, aerated emulsion, to a solid, porous structure, happens later, allowing sufficient expansion of air cells before the batter sets and an increase in cake volume [23,24]. The complex modulus G* during heating of the different batters is presented in Figure 4. Initially, in the 25 to 55/60 °C temperature range, G* increased (e.g., control batter went from 8.29 Pa at 25 °C to 200.79 Pa at 55 °C), which could be attributed to protein–protein interactions [25] and to the presence of the emulsifiers in the cake premix that promoted air incorporation during mixing and retention, giving place to higher batter viscosity throughout the heating process [23]. Further increase of the temperature up to 85–90 °C led to a drop in G* due to sugar dissolution resulting in aqueous phase increase too (G* values at 85 °C: control 58.36 Pa, RS Orafti 77.24 Pa and RS Fibruline 54.45 Pa). Similar observations were reported by Shelke et al. [23] who evaluated batter viscosity during heating. In their study, they reported that not all of the sugar was dissolved in the cake batter prior heating, so the batter contained a higher ratio of solids at ambient temperature. The temperature increase on baking caused higher sugar dissolution, resulting in a greater total volume and reduced viscosity. The RS Fibrulin batter showed lower G* values in this temperature range (60−90 °C) than the other two batters. This behaviour may be related to differences in water binding properties among formulations. According to Hager et al. [26], inulin molecules bind large amounts of water by hydrogen bonds, but this varies with inulin molecular weight. Inulin with lower DP exhibits higher water-binding ability due to lots of external hydroxyl groups and a certain amount of small sugars like glucose, fructose and sucrose [27]. Fibrulin^®^ Instant would have had less interaction with water than Orafti^®^ HSI or sugar (control) because its higher DP, resulting in lower complex modulus values during this temperature range (60–90 °C), the range at which sugar becomes dissolved and other particles (inulin, fructans, starch, proteins) start to hydrate.

Then G* values increased when temperature was above 85–90 °C (second inflexion point of the curves); this change is related to an increase in batter consistency because of starch gelatinisation and protein coagulation processes [24]. Further increase of the temperature above the TST increases the rigidity of the cake (continuous increase of G*) due to water evaporation, until crumb formation occurs. The TST for each formulation was taken as the second inflexion point of the curves and is presented in Table 1. RS Fibruline had significantly lower (*p* < 0.05) TST value (80.8 °C) compared to control (88.5 °C) and RS Orafti (90.2 °C); these latter two samples had a similar (*p* > 0.05) TST value. Water availability in the system versus interactions with components (sugar, starch, protein, etc) defines the TST of the system. Sugar delays starch gelatinisation because it competes with starch and protein for the available water in the system. Moreover, sugar molecules may form bridges between neighbouring polysaccharides located within the amorphous region of the granules and restrict movement of these areas so that gelatinisation is shifted to higher temperatures [28]. As mentioned above, inulin can also bind water, depending on its DP. Based on our results of fibre analysis, Orafti^®^ HSI had a greater amount of short chain FOS (DP3-10) and monosaccharide content (21%) compared to Fibruline^®^ Instant (13%). Therefore, Orafti^®^ HSI would have bound more recipe water and, hence, reduced the water availability for interactions with starch granules and proteins; this may have delayed the thermal setting in RS Orafti batter in comparison with RS Fibruline batter.

#### 3.2.4. Water Loss during Baking, Cake Water Activity (aw) and Moisture Content of Crumb

Water loss during baking did not significantly differ (*p* = 0.101) between formulations (Table 2). Crumb moisture content was similar for both RS samples; control cake exhibited a lower (*p* < 0.05) moisture content than RS Orafti cakes. RS Fibruline cakes had a significantly higher (*p* < 0.05) aw (0.924) compared to control (0.915) and RS Orafti cakes (0.909) (Table 2). As explained in the section above (Section 3.2.3), Fibruline^®^ Instant has a greater content of higher DP fructans than Orafti^®^ HSI, thus lower water binding capacity; as a result, RS Fibruline cakes had higher water available for reactions and microorganisms to use.

#### 3.2.5. Colour Properties of Cake Crumb

Sugar reduction and inulin addition caused cake crumb colour to be significantly darker (lower L*), more red (lower −a*) and more yellow (higher +b*) (Table 3). This effect could be attributed to the presence of inulin, which contains reducing sugars and, therefore, may have accelerated Maillard browning during baking in comparison to the control cake crumb. The control cake contained exclusively sucrose which is not a reducing sugar, and needs to be first hydrolysed during heating in order to contribute to Maillard reaction. Similar results were previously reported for sugar reduced cakes using polydextrose as a sugar replacer [4] and by Rodriguez-Garcia et al. [29] for cakes with inulin as a fat replacer.

#### 3.2.6. Cake Height and Cellular Structure of the Crumb

**Figure 5:**
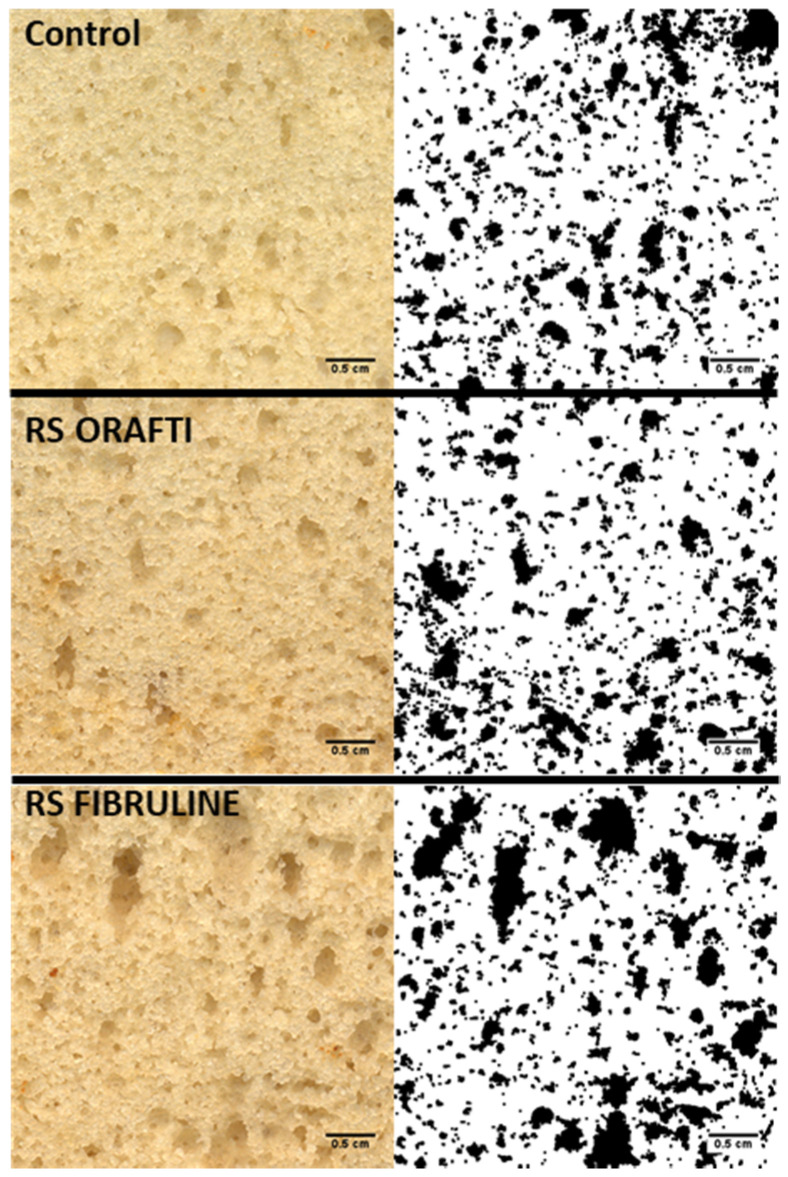
Cellular structure of the crumb of the cakes. **Left column**: scanned crumbs. **Right column**: binarized images of scanned crumbs.

When sugar was replaced by inulin cake height decreased significantly (*p* < 0.05) compared to the control (Table 3). RS Fibruline cake exhibited the lowest height (8.04 cm) (*p* < 0.05) compared to control (9.26 cm) and RS Orafti cake (8.73 cm). Concerning the cell crumb structure, the control cake showed an even distribution of small cells (Figure 5). RS Orafti cake was similar (*p* > 0.05) to the control in terms of mean cell size and cell circularity; however it showed significantly higher (*p* < 0.05) cell density than control and RS Fibruline cakes. 

RS Fibruline cake crumb showed a more heterogeneous distribution of cell, with bigger cells and compact areas in the crumb (Figure 5); this was noted through higher mean cell size and lower circularity values. This finding could be partially attributed to the lower G* during heating (60–90 °C; G* values at 80 °C: control 83.86 Pa, RS Orafti 97.45 Pa and RS Fibruline 54.96 Pa), and lower TST (control 88.5 °C, RS Orafti 90.2 °C, RS Fibruline 80.8 °C) (Table 1) of RS Fibruline batter. On one hand, a lower complex modulus during heating could have given place to air phase instability, so air bubbles could have migrated, coalesced, and were lost before the batter set. On the other hand, an early crumb setting could reduce the time for air nuclei expansion during baking leading to lower cake height.

#### 3.2.7. Textural Properties of Cakes

RS Orafti cake exhibited significantly lower (*p* < 0.05) firmness (5.36 N) compared to control (6.92 N) (Table 3). RS Fibruline cake showed significantly higher (*p* < 0.05) firmness value (7.37 N) than RS Orafti. Although RS Fibruline was firmer than control, there was not a significant difference (*p* > 0.05) among these two samples. The higher firmness values for RS Fibruline cake could be explained by its lower height and less aerated crumb structure. Ronda et al. [30] also found significantly higher firmness in 30% sugar reduced cakes with a mixture of Fibruline^®^ S20 (a high soluble inulin from Cosucra), water and Stevia. In contrast, the significant lower firmness of RS Orafit cake than control could be a result of its higher moisture content compared to control. Struck et al. [5] stated that short-chain inulin shows a higher solubility and a higher water retention capacity compared to sucrose; it does not recrystallise which leads to a softer crumb texture, as we have observed in RS Orafti samples. Similar results have been found in literature when sugar was replaced in cake and muffin formulations by sucralose/polydextrose mixture (25, 50 and 75% sugar replacement) [31], by oligofructose (0, 20, 30, 40 and 50% sugar replacement) [32], by polydextrose, water and Stevia (30% sugar replacement) [30], by erythritol (100% sugar replacement) [33], by a mixture of polydextrose, water and stevia (30% sugar replacement) or by a mixture of wheat bran, water and stevia (30% sugar replacement) [34].

Springiness was significantly higher (*p* < 0.05) for the control sample compared to sugar reduced formulations. Among the sugar reduced formulations, springiness did not present significant differences (*p* > 0.05). These results are in agreement with previous studies with oligofructose as a sugar replacer in cakes [32] and with sucralose/polydextrose mixture as sugar replacer in muffins [31]; they observed that a decrease in springiness was associated with a denser matrix. Both RS cakes showed lower height than control, and RS Fibruline cake showed also lower cell density than control (Table 2). The lower values of springiness for sugar reduced cakes could signify a lower recovery during manipulation which means that these samples are easier to damage and deform. According to Luo et al. [35], inulin enriched steam bread presented lower springiness and recovery values compared to the control sample and this reduction was dependent on the inulin DP. Inulin of higher DP decreased the springiness to a higher extent. In our experiments, this trend was not so obvious; although this is perhaps due to the narrower distribution of DP in comparison to the fibres used in the Luo et al. study [35].

#### 3.2.8. Sensory Profiling of Cakes

The mean results from the sensory profiling of cakes are given, along with significance levels from ANOVA, in Table 4. In terms of appearance attributes, the parameters found to be significantly different among samples were golden colour, dry appearance (approaching significance *p* = 0.055), springiness and dryness to touch, crumbliness, and presence of uneven colour patches. Addition of Orafti^®^ HSI significantly darkened the colour of the cakes, confirming that the differences obtained by the instrumental colour measurements were perceivable by panellists. Furthermore, the addition of the Orafti^®^ HSI increased the number of colour patches in the cake crumb. This finding could be possibly ascribed to the fact that inulin was not completely solubilised, thus, creating crumb spots that caramelise during baking.

RS Orafti cake’s dry appearance, dryness and springiness when touched were not significantly different (*p* > 0.05) from control. On the other hand, RS Fibruline cake was found to be significantly drier, less springy and crumblier compared to control and RS Orafti. The size of bubbles and the variation of the bubble size were not affected by the different fillers used.

Regarding aroma attributes, the control sample exhibited significantly higher milky and eggy aromas compared to RS Orafti cake. On the other hand, RS Orafti cakes showed a significantly higher toasty and buttery notes, while RS Fibruline sample did not differ significantly from the control.

Regarding taste, both sugar reduced cakes were found to be significantly less sweet (*p* < 0.05) compared to the control and there was not significant difference in sweetness level between them. The flavour trends were the same as for aroma, RS Orafti cake received significantly higher scores for toasty flavour compared to control and RS Fibruline cake and was significantly lower in milky and eggy flavour.

Most of the mouthfeel attributes scored did not differ significantly (*p* > 0.05) between formulations. The exception was the perception of the dryness in the mouth during chewing and the extent to which the product coats the mouth during chewing, which were significantly higher (*p* < 0.05) for RS Fibruline cakes. This finding is comparable to the instrumental textural measurements of springiness. More specifically, RS Fibruline cake exhibited significantly lower values of springiness compared to the control. Low springiness values indicate that the sample had a low recovery during mastication; this mechanical behaviour led to samples that were more easily damaged and deformed and more likely to stick to the teeth and coat the mouth during mastication.

Concerning the aftereffects, the feeling of sweet taste lasting longer (“builds sweetness”) was significantly higher (*p* < 0.05) for the control sample compared to the sugar reduced formulations. This may reflect the higher capacity of sugar to build sweetness during chewing than the replacers in the control sample rather than a real difference in temporal profile. The eggy and milky aftereffects were significantly lower for the RS Orafti sample and buttery and toasty remained significantly higher as aftereffects, while the feeling of dryness after swallowing was significantly higher for RS Fibruline cake.

### 3.3. The Influence of Inulin as a Sugar Replacer in Semi-Sweet Hard Dough Biscuits

#### 3.3.1. Viscoelastic Properties of Biscuit Doughs

**Figure 6:**
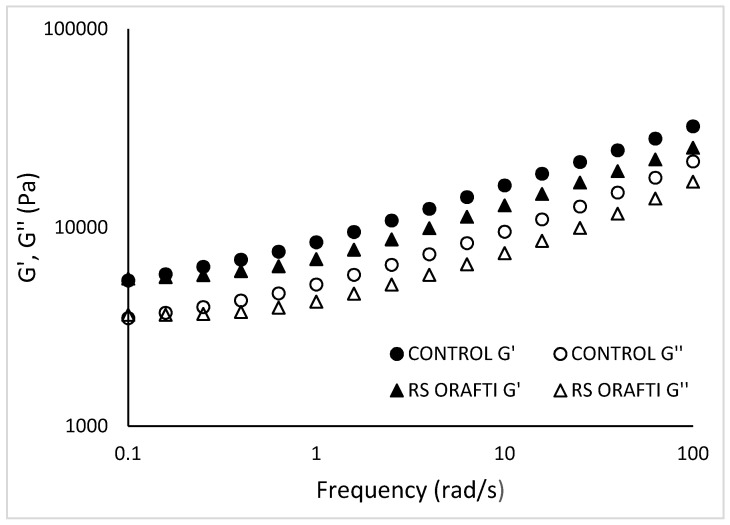
Frequency dependence of the storage G′ and loss G″ modulus of the various doughs. Control (full sugar dough biscuit), RS Orafti (30% sugar reduced dough biscuit with Orafti^®^ HSI inulin). The data are mean values of replicates.

**Figure 7:**
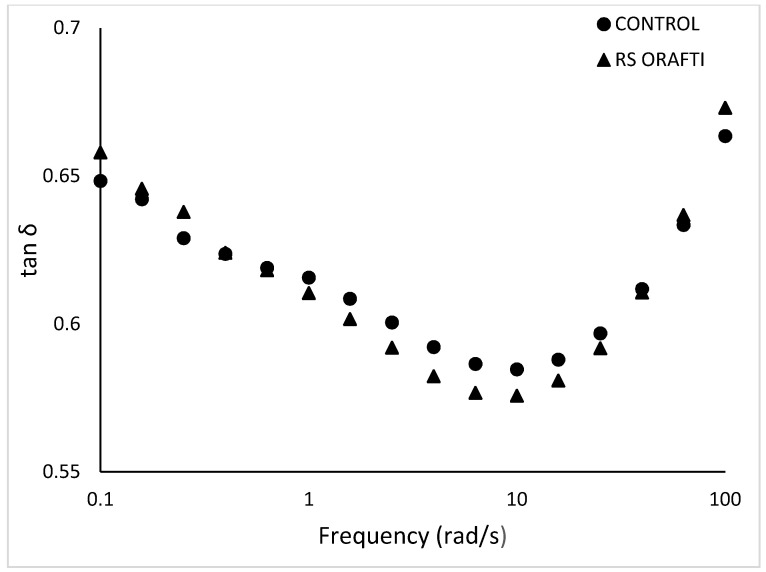
Frequency dependence of the tanδ of the various doughs. Control (full sugar dough biscuit), RS Orafti (30% sugar reduced dough biscuit with Orafti^®^ HSI inulin). The data are mean values of replicates.

The viscoelastic properties of the biscuit dough were studied by frequency sweep tests (Figure 6 and Figure 7). The values for G′ and G″ were, as expected, much higher for biscuit doughs (control dough at 10 rad/s: G′ 16,285.17 Pa, G″ 12,926.70 Pa) than for cake batters(control batter at 10 rad/s: G′ 175.79 Pa and G″ 149.72 Pa); in addition, G′ was more clearly separated from G″ (Figure 6) for doughs than for batters (Figure 3, control and RS Orafti dough samples), indicating a more viscoelastic liquid-like behaviour of batters and a more viscoelastic solid-like behaviour of biscuit dough.

For all the dough samples, the elastic component G′ was higher than the viscous component G″ indicating a solid like behaviour. Increasing frequency increased both G′ and G″. The mechanical properties of RS Orafti dough were relatively similar to the control. Inulin generally has higher affinity for water compared to sucrose, taking up more water and leaving less water available for gluten development [36,37]. Gluten development contributes to the elasticity of the dough, therefore, sugar allows greater gluten development and a more elastic dough compared to inulin. The biscuits in this study were hard dough biscuits where the gluten network should be developed up to a certain extent. Therefore, if the measured difference in dough properties (Figure 6 and Table 5) led to a perceptual difference, then a softer biscuit would result (see Table 6). Dependence of tan δ vs. frequency range is shown in Figure 7. At low frequencies, tan δ decreased when frequency was increased; at high frequencies (>10 rad/s) tan δ increased as frequency increased too. These differences imply that the dough acted more like a solid when imposed to slow changes in stresses, but very fast changes in applied stress will make the dough act more as a liquid. Similar rheological behaviour was reported from other researchers for cookie dough [38] and biscuit dough [39].

#### 3.3.2. Rheological Properties of Biscuit Dough during Heating.

**Figure 8:**
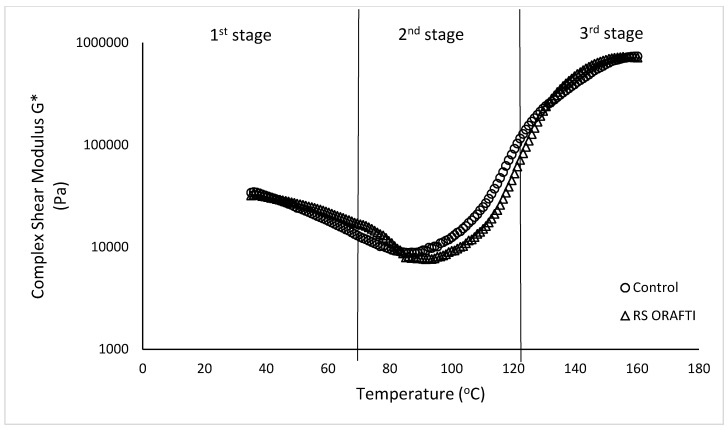
Changes in complex moduli during heating of biscuit dough. Control (full sugar dough biscuit), RS Orafti (30% sugar reduced dough biscuit with Orafti^®^ HSI inulin).

Three distinct stages of moduli build-up were observed during baking for all the dough samples: (1) bubble growth, (2) rapid expansion/starch gelatinization and (3) final curing (Figure 8). In the first stage, G* values decreased as the dough temperature increases from 35 to 80–90 °C (e.g., control dough went from 31,333.00 Pa at 40 °C to 9349.70 Pa at 90 °C). This decrease could be ascribed to fat melting and to the activity of the leavening agents. The release of CO_2_ causes bubble expansion and leads to a decrease in dough consistency. Further increase of the dough temperature causes an increase in the complex modulus of the dough due to starch gelatinisation and protein denaturation (second phase 80–150 °C; Control 706,786.67 Pa and RS Orafti 724,833.33 Pa). Evaporation of water with increased temperature further stiffens the dough. In the third stage (>150 °C), the dough reaches its final plateau modulus and is a cellular solid with open cells [40].

The TST of the hard dough biscuits is presented in Table 5. RS Orafti dough exhibited no significant difference in the TST compared to control dough; similar results were found for cake batters (Table 1). These results suggested that Orafti^®^ HSI has similar water binding capacity to sugar.

#### 3.3.3. Textural Properties of Biscuit Doughs

The effect of sugar replacement on the texture of the biscuit doughs is shown in Table 5. The control was found to be the softest dough (*p* < 0.05) (2.46 N), while the sugar replaced formulation exhibited an increase in hardness (2.77 N).

Stickiness was found to be significantly lower for the sugar replaced formulation. Dough stickiness is defined by the interaction of two forces: the adhesive force (interaction between material and a surface) and the cohesive force (internal rheological properties of the material) [41]. Orafti^®^ HSI inulin would have a higher water holding capacity compared to sucrose in the biscuit dough system; similar observations were made previously for inulin in comparison to sucrose in sugar reduced formulations [36,37]. The reduced availability of water in the inulin containing dough accounts for their lower stickiness and is supported by their lower aw (Table 6).

#### 3.3.4. Water Activity (aw) and Moisture Content of Biscuit Crumb

The aw of the samples and the moisture content are presented in Table 6. The aw values were in line with those expected of commercial biscuits (0.1–0.23; [42]). Control biscuit showed significantly higher aw (0.175) than RS Orafti biscuit (0.161), which is in-line with the higher stickiness of the control dough (Table 5).

Biscuits are very low moisture content products; the majority of the moisture lies in a thin lamella of material near the centre, while the surface and the outer periphery of the product are nearly dry. The typical initial moisture content of biscuit dough ranges from about 11–30%, comprising both added water at the dough mixing stage and water naturally occurring in the ingredients. During the baking step, the final moisture content reduces to 1–5% in the final product. [43]. In our study, no significant differences in moisture were observed among full sugar (1.91%) and sugar reduced biscuits (2.01%).

#### 3.3.5. Colour Properties of Biscuits

RS Orafti biscuits were not significantly different in colour compared to the control (Table 6). Unlike with the cake products, the use of inulin as a sugar replacer did not significantly affect the L*, a*, b* values. Our results were in agreement with Gallagher et al. [3], who replaced sugar with oligofructose in short dough biscuits and reported that the resulting biscuits had a similar brown colour to the ordinary sugar biscuits.

#### 3.3.6. Dimensions and Textural Properties of Biscuits

There were no differences in biscuit dimensions between the RS Orafti and control biscuits (Table 6). Sucrose acts as a hardening agent by crystallizing as the biscuit cools, making the product crisp. Water is limited in the biscuit dough, so sugar dissolution is so slow that local sugar concentrations remain high, which leads to sugar recrystallisation during baking [44]. Significantly lower hardness levels (*p* < 0.05) were found for RS Orafti biscuit (8.23 N) compared to control (15.25 N) (Table 6). This finding was in line with expectation as the inulin cannot crystallise after baking. Handa et al. [37] reported that short dough cookies enriched with FOS, had lower hardness as compared to control sucrose cookies. Similarly, Gallagher et al. [3] found significantly lower hardness levels for short dough biscuits, when replacing sugar with raftilose (inulin) up to 30%.

#### 3.3.7. Sensory Profiling of Biscuits

The mean results from the sensory profiling of biscuits are given, along with significance levels from ANOVA, in Table 4.

The only appearance attribute found to be significantly different between the samples was density of crumb; the RS Orafti biscuit had a less dense (more aerated) crumb than the control. Although this sensorial difference was not large enough to have caused any difference in biscuit dimensions, it could relate to the softer texture of sugar replaced biscuits (Table 6).

There were no significant differences in aroma, taste or after-effects attributes between control and RS Orafti biscuits. Regarding taste, the 30% reduction in sugar led to no significant difference in sweetness. This is perhaps because the biscuit was a hard dough biscuit type containing 22% sugar initially and the total sugars reduction to 15% was below the limits of a just noticeable difference for sweetness in this matrix. However, there was a significant difference in perception of vanilla flavour, with the RS Orafti scoring lower than the control. The mean values for vanilla flavour were low in both biscuit types and there was no vanilla flavour added to the dough; as vanilla is closely associated with the perception of sweetness, this small difference may reflect a slight reduction in sweetness overall in the sugar reduced biscuit.

In terms of the mouthfeel attributes, as anticipated from the physical analysis (Table 6), RS Orafti biscuits were perceived significantly softer and less crunchy than the control biscuit (Table 4), as well as less dry. These results could be related to the perception of the sugar reduced biscuits as more aerated. These differences in perceived texture result from the use of inulin to replace sugar which has caused a change in water binding, gluten development and recrystallisation, as discussed above.

## 4. Conclusions

In cakes the use of an inulin with lower DP (Orafti^®^ HSI) led to physical parameters of batter and cake more similar to the control than when using a higher DP inulin (Fibruline^®^ Instant). The baked RS Orafti cake was significantly less firm than the control cake, but this difference was not substantial enough to be noticed by the sensory panel. This cake had a noticeably more golden colour. RS Fibruline cake had a lower height, higher springiness and dryness on tasting than the control, confirming that the DP of the inulin used is crucial to the success of using inulin as a sugar replacer in cakes. The sugar replaced cakes were significantly less sweet and had some significant differences in aroma and flavour compared to the control product. If the intention is to maintain sweetness in the sugar replaced products, then a solution to increasing the sweet taste would need to be found in addition to using the inulin to replace the bulking and physical properties of the sugar.

The use of the low DP inulin in hard-dough biscuits (Orafti^®^ HSI) led to a less hard and less crunchy product than the control biscuit, due to lower sugar recrystallization during baking. Regarding sweetness, at this 30% reduction in sugar (from 22% to 15% total sugar in the product) the change in sugar content did not lead to a reduction in biscuit sweetness as measured by sensory profiling. This suggests that, if the changes in texture are acceptable to consumers, higher levels of sugar reduction could be aimed for in this type of hard-dough biscuit.

## Figures and Tables

**Table 1:** Cake batter properties: Rheology (Power law model; K consistency index; n flow behavior index) and thermal setting temperature (TST). Control (full sugar cake batter), RS Orafti (30% sugar reduced cake batter with Orafti^®^ HSI inulin), RS Fibruline (30% sugar reduced cake batter with Fibruline^®^ Instant inulin).

Cake Batter	K (Pa·s^n^)	n (-)	R^2^	TST (°C)
Control	18.48 ^b^ (3.85)	0.58 ^a^ (0.03)	0.99	88.5 ^a^ (0.6)
RS Orafti	20.79 ^b^ (2.54)	0.59 ^a^ (0.03)	0.99	90.2 ^a^ (1.4)
RS Fibruline	130.09 ^a^ (11.41)	0.39 ^b^ (0.01)	0.99	80.8 ^b^ (2.1)

In parentheses standard deviation values. Samples with different letters in the same column differ significantly (*p* < 0.05). ‘K’ is the consistency index and ‘n’ is the flow behaviour index.

**Table 2:** Cake properties: Mean values of water loss during baking (WL), moisture content and water activity (aw). Control (full sugar cake batter), RS Orafti (30% sugar reduced cake batter with Orafti^®^ HSI inulin), RS Fibruline (30% sugar reduced cake batter with Fibruline^®^ Instant inulin).

Cake	WL (%)	Moisture Content (%)	aw
Control	8.70 ^a^ (0.56)	29.39 ^b^ (0.29)	0.915 ^b^ (0.010)
RS Orafti	9.39 ^a^ (0.24)	30.60 ^a^ (0.27)	0.909 ^b^ (0.003)
RS Fibruline	8.90 ^a^ (0.65)	30.38 ^ab^ (0.22)	0.924 ^a^ (0.006)

In parentheses standard deviation values. Samples with different letters in the same column differ significantly (*p* < 0.05).

**Table 3:** Cake properties: Mean values of colour parameters, cake height and crumb cellular characteristics, firmness, and springiness. Control (full sugar cake), RS Orafti (30% sugar reduced cake with Orafti^®^ HSI inulin), RS Fibruline (30% sugar reduced cake with Fibruline^®^ Instant inulin).

Cake	L*	a*	b*	Height (cm)	Cell Area (mm^2^)	Cell Circularity	Cell Density (cells/cm^2^)	Firmness (N)	Springiness (%)
Control	73. 4 ^a^ (0.87)	−2.58 ^b^ (0.20)	20. 5 ^c^ (0.72)	9.26 ^a^ (0.32)	0.64 ^b^ (0.10)	0.79 ^a^ (0.01)	30 ^b^ (5)	6.92 ^a^ (0.45)	48.5 ^a^ (1.4)
RS Orafti	70.4 ^b^ (0.61)	−0.93 ^a^ (0.08)	24.7 ^a^ (0.31)	8.73 ^b^ (0.25)	0.67 ^ab^ (0.09)	0.78 ^ab^ (0.01)	31 ^a^ (2)	5.36 ^b^ (0.29)	46.5 ^b^ (1.5)
RS Fibruline	70.4 ^b^ (0.83)	−1.05 ^a^ (0.10)	22.8 ^b^ (0.89)	8.04 ^c^ (0.43)	0.79 ^a^ (0.10)	0.77 ^b^ (0.01)	29 ^b^ (3)	7.37 ^a^ (0.66)	46.1 ^b^ (1.8)

In parentheses standard deviation values. Samples with different letters in the same column differ significantly (*p* < 0.05).

**Table 4:** Mean values of sensory attributes for cakes and biscuits prepared with standard sugar and reduced sugar content. Cakes samples: Control (full sugar cake), RS Orafti (30% sugar reduced cake with Orafti^®^ HSI inulin), RS Fibruline (30% sugar reduced cake with Fibruline^®^ Instant inulin). Biscuit samples: Control (full sugar hard dough biscuit), RS Orafti (30% sugar reduced hard dough biscuit with Orafti^®^ HSI inulin).

	Cake Samples				Biscuit Samples		
Attribute	Control	RS Orafti	RS Fibruline	Sample Significance (*p*-Value)	Control	RS Orafti	Sample Significance (*p*-Value)
**Appearance**							
Golden colour	17.6 ^b^	56.2 ^a^	25.0 ^b^	<0.0001	35.6	45.5	0.106
Size of bubbles	35.2	31.7	30.5	0.334			
Variation in bubble size	46.2	47.0	53.5	0.317			
Dry appearance	40.2 ^b^	39.9 ^b^	53.8 ^a^	0.055			
Springiness to touch	43.3 ^a^	52.0 ^a^	29.8 ^b^	0.0007			
Firm to Touch	50.4	51.3	59.8	0.161			
Dry to touch	32.0 ^b^	31.7 ^b^	45.2 ^a^	0.0036			
Crumbly when pulled apart	17.7 ^b^	13.1 ^c^	45.3 ^a^	<0.0001			
Uneven colour patches	0.4 ^b^	22.4 ^a^	0.6 ^b^	0.0001			
Uneven top surface					7.7	8.8	0.499
Density of the crumb at the cross section					63.5 ^a^	48.0 ^b^	0.048
Uniformity of the surface colour					53.9	52.9	0.854
**Aroma**							
Milky	29.4 ^a^	18.1 ^b^	25.8 ^ab^	0.034	19.6	17.7	0.260
Toasty	7.9 ^b^	31.8 ^a^	9.6 ^b^	0.002			
Buttery	9.4 ^b^	21.3 ^a^	13.0 ^ab^	0.052	13.2	12.9	0.930
Sweet	36.2	37.3	36.9	0.927	32.0	34.4	0.569
Eggy	23.4 ^a^	9.9 ^b^	18.4 ^ab^	0.015			
Musty	5.5	3.0	8.0	0.159			
Caramel					19.1	19.5	0.927
Vanilla					16.1	10.9	0.089
Degree of baked note					32.2	32.6	0.918
**Taste-flavour**							
Sweet	50.0 ^a^	38.4 ^b^	42.2 ^b^	0.001	30.4	32.8	0.354
Salty	3.6	4.6	4.6	0.394	4.4	4.9	0.536
Buttery	10.7	19.3	13.1	0.097	13.9	12.2	0.370
Milky	25.7 ^a^	14.6 ^b^	21.5 ^a^	0.002	18.0	16.5	0.446
Toasty	4.9 ^b^	21.2 ^a^	7.9 ^b^	0.002			
Eggy	17.1 ^a^	8.9 ^b^	13.2 ^ab^	0.023			
Vegetable oil	10.7	8.7	7.9	0.089			
Vanilla					14.1 ^a^	8.4 ^b^	0.011
Caramel					12.7	15.6	0.491
Degree of Baked note					34.9	34.0	0.788
**Mouthfeel**							
Hardness of first bite	27.2	28.8	25.9	0.879	72.7 ^a^	59.3 ^b^	0.013
Rate of dispersion (Dissolving)	45.0	44.5	43.2	0.900	43.4	38.1	0.174
Dryness	29.5 ^b^	34.3 ^b^	53.4 ^a^	0.0001	68.9	58.8	0.0005
Mouth coating	35.4 ^b^	36.7 ^b^	45.5 ^a^	0.048			
Body (dense on chewing)	43.2	44.3	44.1	0.978			
Salivating	29.9	30.7	32.1	0.858	34.6	32.2	0.409
Cooling	4.8	7.3	6.2	0.269	2.1	3.6	0.310
Crunchy					74.4 ^a^	63.7 ^b^	0.009
Tooth packing					55.0	54.3	0.839
Cohesiveness—forms bolus					22.2	25.3	0.185
Tingling sensation					2.3	3.8	0.307
Tongue numbing					2.1	4.7	0.210
**Aftereffects**							
Builds sweetness	42.8 ^a^	31.6 ^b^	33.8 ^b^	0.032	21.8	23.9	0.374
Metallic	11.3	7.6	8.0	0.409			
Bitter	4.9	5.6	5.6	0.831	3.5	3.8	0.742
Eggy	12.0 ^a^	6.0 ^b^	11.5 ^a^	0.007			
Milky	18.1 ^a^	10.8 ^b^	16.0 ^a^	0.003			
Buttery	6.5	14.7	9.0	0.066	6.1	6.7	0.808
Toasty	4.5 ^b^	14.9 ^a^	5.4 ^b^	0.0003			
Cooling	4.9	5.6	5.8	0.803	3.0	3.4	0.461
Throat catch	4.5	2.4	6.4	0.478			
Dryness	30.2 ^b^	29.6 ^b^	44.0 ^a^	0.026			
Salivating	34.6	30.1	35.0	0.452	22.9	26.1	0.347
Vanilla					7.7	6.0	0.231
Caramel					10.0	11.7	0.479
Degree of baked note					21.7	22.5	0.658
Salty					3.9	3.8	0.922
Tooth packing					34.9	33.2	0.561
Tingling sensation					4.1	4.1	0.945
Tongue numbing					3.4	4.9	0.397

Values with the same superscript letter in the row (within cakes, or within biscuits) did not differ significantly (*p* < 0.05). Where there is no data in a cell it relates to an attribute that was not measured in that matrix (i.e., cake or biscuit).

**Table 5:** Biscuit dough: Thermal setting temperature (TST), firmness and stickiness. Control (full sugar dough), RS Orafti (30% sugar reduced dough with Orafti^®^ HSI inulin).

Biscuit Dough	TST (°C)	Hardness (N)	Stickiness (N)
Control	93.76 ^a^ (1.90)	2.46 ^b^ (0.2)	0.59 ^a^ (0.08)
RS Orafti	93.46 ^a^ (4.62)	2.77 ^a^ (0.3)	0.54 ^b^ (0.05)

In parentheses standard deviation values. Samples with different letters in the same column differ significantly (*p* < 0.05).

**Table 6:** Biscuit parameters: Water activity (aw), moisture content, colour and hardness. Control (full sugar hard dough biscuit), RS Orafti (30% sugar reduced hard dough biscuit with Orafti^®^ HSI inulin).

Biscuit	aw	Moisture Content (%)	L*	a*	b*	Hardness (N)	Length (mm)	Width (mm)	Thickness (mm)
Control	0.175 ^a^ (0.005)	1.92 ^a^ (0.27)	76.3 ^a^ (2.12)	2.39 ^a^ (1.80)	31.45 ^a^ (2.13)	15.25 ^a^ (2.70)	69.7 ^b^ (0.70)	54.6 ^a^ (1.01)	4.8 ^a^ (0.52)
RS Orafti	0.161 ^b^ (0.009)	2.01 ^a^ (0.20)	75.6 ^a^ (2.62)	3.16 ^a^ (1.81)	32.98 ^a^ (2.25)	8.23 ^b^ (0.62)	69.7 ^a^ (0.78)	55.3 ^a^ (0.56)	4.5 ^a^ (0.30)

In parentheses standard deviation values. Samples with different letters in the same column differ significantly (*p* < 0.05).

## Data Availability

The data presented in this study are openly available in the University of Reading Research Data Archive at http://dx.doi.org/10.17864/1947.292 (accessed on 25 February 2021).

## Supplementary Materials

The following are available online at https://www.mdpi.com/article/10.3390/foods10050951/s1, Table S1: Descriptive vocabulary and definitions used by trained panelists to evaluate cakes.
