# Supplementary material for: The Functionality of Inulin as a Sugar Replacer in Cakes and Biscuits; Highlighting the Influence of Differences in Degree of Polymerisation on the Properties of Cake Batter and Product
Source: Foods. 2021 Apr 27;10(5):951. doi: 10.3390/foods10050951 (PMC8146277; doi:10.3390/foods10050951)
Supplement: Supplementary file 1 [file foods-10-00951-s001.zip › foods-1151957-supplementary.pdf]

Table S1. Descriptive vocabulary and definitions used by trained panelists to evaluate cakes.

Attribute	Definition / Reference	Anchors
Appearance		
Golden colour	Crumb colour ranging from light golden to dark golden	Light to Dark
Size of bubbles	The bubble size ranging from small to large	Small to Large
Variation in bubble size	Where some cakes are very even in bubbles size, other cakes may vary from small bubbles to very large bubbles	Very Little to Lots
Dry appearance	Cake surface appears dry or moist	Moist to Dry
Springiness to touch	Spongy when pressed with finger	Not to Very
Firm to touch	Firmness when pressed with finger	Not to Very
Dry to touch	The perception of dryness when touching the sample	Moist to Dry
Crumbly when pulled apart	The extent to which the sample falls apart into small pieces when broken into two by hand	Not to Very
Uneven colour parches	The amount of visual uneven colour patches when cake broken into two by hand	None to Lots
Aroma		
Milky	Cooked Milk aroma (UHT Milk)	Not to Very
Buttery	Melted butter aroma	Not to Very
Toasty	Aroma of toasted white bread	Not to Very
Sweet	Aroma of demerara sugar	Not to Very
Eggy	Aroma of scrambled egg whites	Not to Very
Musty	Aroma of staled white bread	Not to Very
Taste -flavour		
Sweet	Taste of sucrose (sugar) solution	Low to Strong

		Standards were given as 5 anchors along the line scale
Salty	Taste of salt (sodium chloride) solution	Not to Very
Milky	Flavour of cooked milk	Not to Very
Buttery	Flavour of melted butter	Not to Very
Toasty	Flavour of toasted white bread	Not to Very
Eggy	Flavour of scrambled egg whites	Not to Very
Vegetable oil	Flavour of vegetable (rapeseed oil)	Not to Very
Mouthfeel		
Hardness of first bite	The force perceived when biting into cake	Soft to Hard
Rate of dispersion (dissolving)	The rate at which the sample breaks down in the mouth	Slow to Quick
Dryness	Cake feels moist or dry in the mouth during chewing	Moist to Dry
Mouthcoating	The extent to which the product coats the mouth	Not to Very
Body (dense on chewing)	How dense the product feels on chewing	Light to Dense
Salivating	The amount of saliva production during product chewing	Not to Very
Cooling	The sensation of reduced temperature experienced during product chewing	Not to Very
Aftereffects		
Builds sweetness	The extent to which sweetness lasts after swallowing the product	Not to Very
Metallic	Taste of iron solution	Not to Very
Bitter	Taste or bitter (quinine) solution	Not to Very
Eggy	As for flavour	Not to Very
Milky	As for flavour	Not to Very

Buttery	As for flavour	Not to Very
Toasty	As for flavour	Not to Very
Cooling	As for mouthfeel	Not to Very
Throat catch	The extent to which the swallowing of the sample causes an unpleasant feeling- mild pain in the throat	Not to Very
Dryness	The feeling of moisture or dryness after swallowing of the sample	Moist to dry
Salivating	The amount of saliva production after swallowing the cake	Not to Very

Supplementary Table S2 Descriptive vocabulary and definitions used by trained panelists to evaluate biscuits

Attribute	Definition / Reference	Anchors
Appearance		
Golden colour- top surface	Degree of surface colour darkness	Light golden to dark golden
Uneven top surface	The presence of uneven points on the top surface - Cracks and wrinkles. Even (absence of uneven places on the surface)	Even to rough
Density of the crumb at the cross section	The density of the crumb	Aerated to compact (dense)
Uniformity of the surface colour	The colour difference between the edges and the main surface (presence of darker edges - not uniform)	Not to Very
Aroma		
Sweet	Demerara sugar	Not to Very
Buttery	Melted butter	Not to Very

Caramel	Caramel syrup	Not to Very
Vanilla	Vanilla extract (Waitrose)	Not to Very
Milky	Cooked Milk aroma (UHT Milk)	Not to Very
Degree of baked note	Ice cream waffle cones	Low to Strong
Taste -flavour		
Sweet	Fundamental taste sensation typical of sucrose	Low to Strong<br><br>Standards will be given as 5 anchors along the line scale
Vanilla	The same as aroma but perceived orally	Not to Very
Buttery	The same as aroma but perceived orally	Not to Very
Milky	The same as aroma but perceived orally	Not to Very
Caramel	The same as aroma but perceived orally	Not to Very
Degree of Baked note	The same as aroma but perceived orally	Low to Strong
Mouthfeel		
Hardness of first bite	The force perceived when biting the biscuit	Soft to hard

Crunchy	The product is hard to break down (high level of force) and divides when chewed with the back molars, while produces a loud (characteristic) noise/sound, ranging from not to very.	Not to Very
Tooth packing	Degree to which the biscuit sticks to the surface of teeth	Not to Very
Cohesiveness – forms bolus	Degree to which bolus holds together in a mass during chewing	Not to Very
Dissolving rate	The time that the biscuit takes to dissolve in the mouth	Quick to Slow
Dryness	The feeling of dryness in the mouth during chewing ranging	Moist to Dry
Salivating	The amount of saliva production during product mastication	Not to Very
Cooling	The sensation of reduced temperature experienced during product mastication	Not to Very
Tingling sensation	A feeling of tingling on the tongue while chewing	Not to Very
Tongue numbing	A feeling of loss of sensation on the tongue while chewing	Not to Very
Aftereffects		
Sweet	As for flavour	Not to Very
Vanilla	As for flavour	Not to Very
Caramel	As for flavour	Not to Very
Degree of baked note	As for flavour	Low to Strong
Buttery	As for flavour	Not to Very

Bitter	The basic taste factor typical of caffeine solution	Not to Very
Salty	The basic taste factor typical of sodium chloride	Not to Very
Cooling	As for mouthfeel	Not to Very
Salivating	The amount of saliva production after swallowing the biscuit	Not to Very
Tooth packing	As for mouthfeel	Not to Very
Tingling sensation	As for mouthfeel	Not to Very
Tongue numbing	As for mouthfeel	Not to Very
